# Antimicrobial Resistance Following Prolonged Use of Hand Hygiene Products: A Systematic Review

**DOI:** 10.3390/pharmacy10010009

**Published:** 2022-01-04

**Authors:** Gouri Rani Banik, Bandar Durayb, Catherine King, Harunor Rashid

**Affiliations:** 1 Sydney Institute for Infectious Diseases, Faculty of Medicine and Health, The University of Sydney, Sydney, NSW 2145, Australia; catherine.king@health.nsw.gov.au (C.K.); harunor.rashid@health.nsw.gov.au (H.R.); 2 The Children’s Hospital at Westmead Clinical School, Faculty of Medicine and Health, The University of Sydney, Sydney, NSW 2145, Australia; 3 Jazan Health Affairs General Directorate, Ministry of Health, Jazan 82611, Saudi Arabia; dr.bander@hotmail.com; 4 Sydney School of Public Health, Faculty of Medicine, The University of Sydney, Sydney, NSW 2006, Australia; 5 National Centre for Immunisation Research and Surveillance (NCIRS), The Children’s Hospital at Westmead, Sydney, NSW 2145, Australia

**Keywords:** antimicrobial agents, antimicrobial resistance, hand hygiene, hand hygiene products

## Abstract

Background: This systematic review aimed to establish whether antimicrobial resistance (AMR) occurs following prolonged use of antimicrobial hand hygiene (HH) products, and, if so, in what magnitude. Methods: Key bibliographic databases were searched to locate items on HH use and AMR development from database inception to December 2020. Records were screened and full texts of all potentially eligible articles were retrieved and checked for inclusion. The following data from the included studies were abstracted: type of HH product used, including the name of antimicrobial agent, study setting, country, study year, duration of use and development of AMR including the organisms involved. Quality assessment was done using the Newcastle-Ottawa Scale (NOS). Results: Of 339 full-text articles assessed for eligibility, only four heterogeneous United States (US) studies conducted in the period between 1986 and 2015 were found eligible, and included. One hospital-based study showed evidence of AMR following long term use of HH products, two studies conducted in household settings showed no evidence of AMR, and another experimental study showed partial evidence of AMR. The overall certainty of the evidence was moderate. Conclusion: Prolonged use of HH products may cause AMR in health care settings, but perhaps not in other settings.

## 1. Introduction

Microbes have become increasingly resistant to common antimicrobial agents. This soaring antimicrobial resistance (AMR) makes it not only harder to treat and prevent infections but has serious cost-implications for public health [1]. Non-judicious use of antimicrobial agents is believed to be the most important trigger for the development of AMR [2]. The fundamental underlying principle is that microbes are able to adapt to defeat stress in their environment, and when repeatedly exposed to traces of antimicrobial agents, they may overcome the pharmacological effects of the agents [3,4]. Antiseptic and antibacterial hand hygiene (HH) products often contain bactericidal, fungicidal and virucidal products, prolonged use of which may lead to development of AMR.

HH refers to the act and process of keeping hands clean and free from germs. HH is considered an essential means of preventing nosocomial infection in healthcare settings [5,6]. HH products usually contain detergents with/or without alcohol, however some manufacturers add antimicrobial compounds with the aim of making the product more effective against pathogens [7]. The use of antimicrobial soaps and other hand washing products is widespread [8,9]; this is especially so following the emergence of SARS-CoV-2 [10], with HH practice being strongly recommended to prevent acquisition and transmission of infection. Active compounds used in HH products vary by brand. While ethanol is predominantly used in most products, synthetic compounds like triclosan, a phenoxyphenol that has microbicidal effects, have also been used [9,11,12]. Triclosan has been widely used in consumer liquid hand soaps to inhibit the growth of both gram-positive and gram-negative bacteria in situ with varying degrees effectiveness. This stresses the microorganisms and may lead to the evolution of a subgroup of microorganisms that are resistant to the antimicrobial compounds that are used in HH products. There is a concern that antimicrobial or antiseptic HH products may lead to the development of AMR, especially if used for prolonged periods and repeatedly [13,14].

Experiments that evaluated the effects of long-term exposure of bacteria to antibacterial agents used in HH products indicated that acquired or intrinsic resistance is possible [15,16,17]; however, there is no focussed systematic review to quantify the magnitude of development of AMR following prolonged use of antimicrobial compounds in HH products. Therefore, we have undertaken a systematic review to establish whether AMR occurs following prolonged use of antibacterial hand washing products, and, if so, with what magnitude of effect.

## 2. Materials and Methods

Key bibliographic databases were searched by an experienced medical information specialist (CK) to locate items on hand sanitiser use and AMR. The databases searched included: OVID Medline all including Epub Ahead of Print, In-Process & Other Non-Indexed Citations, Daily and Versions (1946–6 December 2020), OVID Embase (1974–12 December 2020), CINAHL via EBSCO (1982–December 2020), Cochrane Library databases including Database of Systematic Reviews (Issue 12 of 12 December 2020) and Central Register of Controlled Trials (Issue 12 of 12, December 2020), SCOPUS (1823–December 2020) and Web of Science Core Collection including Science Citation Index Expanded (1900–December 2020), Social Sciences Citation Index (1956–December 2020), Arts & Humanities Citation Index (1975–December 2020), Emerging Sources Citation Index (2015–December 2020), Conference Proceedings Citation Index-Science (1990–December 2020), Conference Proceedings Citation Index-Social Sciences & Humanities (1990–December 2020), Current Chemical Reactions (1985–December 2020) and Index Chemicus (1993–December 2020).

Search terms included thesaurus terms such as ‘Hand’, ‘Hand hygiene’, ‘Anti-Infective Agents, Local’, ‘Surface-Active Agents’, ‘Drug Resistance, Microbial’ as well as relevant associated textword terms. Truncation was used to ensure variant endings of textwords. To minimise bias, no publication date and language limits were used. The systematic review was prospectively registered in PROSPERO (ID: CRD42017070054) at York University, United Kingdom (UK) [18]. Hand searching of the included articles and relevant reviews was carried out to identify additional potential studies.

### 2.1. Inclusion and Exclusion Criteria

The participants of interest were any individuals who use hand washing products containing an antimicrobial agent for a long time. These included community dwellers, household members, health care workers (HCWs) and travellers. Additionally, any in-vitro study looking at the study question, and observational and interventional studies that reported AMR development following the use of specified HH products in household, work, community and healthcare settings were considered. Long-term use was defined as HH use of more than a month. This was decided based on experimental evidence. It is reported that resistance may develop as early as in 10–12 days of microbes’ exposure to antimicrobial agents [19], so a limit of up to one month was considered prolonged for the purpose of our systematic review.

The studies that used/analysed hand washing products for very short duration (<one month) were excluded during the selection of eligible studies. In addition, HH products containing only alcoholic products and no antimicrobial agents or that did not report frequency or pattern of AMR use were excluded.

### 2.2. Study Identification and Data Synthesis

**Figure 1:**
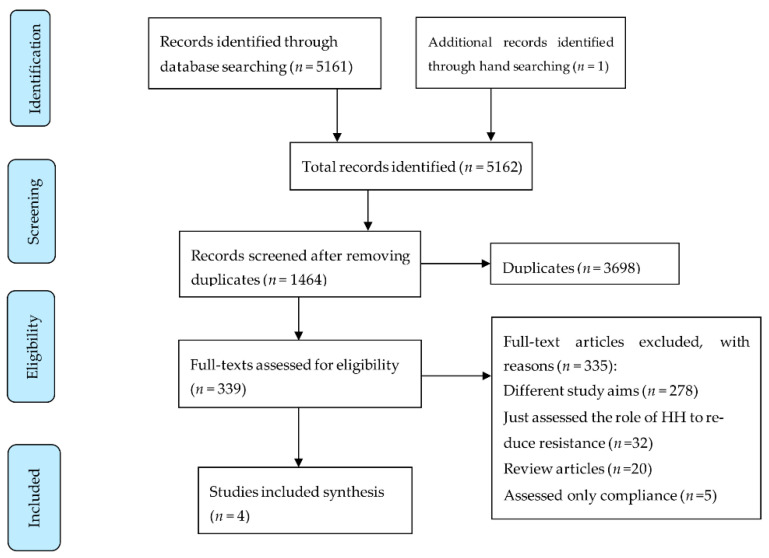
Flow diagram of systematic review searching strategy and included studies.

All records searched from the databases were screened independently by at least two reviewers (GRB, HR). The first reviewer screened all titles, and the second reviewer double checked the screening results. Full texts of all potentially eligible articles were retrieved and checked for inclusion. Data from a total of 339 articles were initially abstracted, and ultimately 4 articles were included (Figure 1). The data were individually extracted in an Excel sheet by three reviewers (GRB, BD, HR). The data abstracted were: type of HH used, compliance rate, study setting, country, year, age of subjects, duration for study, factors affecting compliance, diagnostic method used to establish AMR, and the frequency of detecting AMR organisms. A meticulous review of methodologies was carried out particularly exploring the compounds used in HH products such as antibacterial, antiviral, antifungal, anti-parasitic and antiseptic substances, duration of use, and discomfort reported by consumers. The frequency of AMR was recorded including the profile of resistant organisms. Finally, the study limitations, as acknowledged by the study authors and also as determined by the reviewers, were recorded. All entries were double-checked by two reviewers (GRB, HR); the discrepancies were resolved through regular meetings and discussions.

### 2.3. Quality Assessment

The Newcastle-Ottawa Scale (NOS) was used for assessing the quality of nonrandomised studies. We rated the quality of the studies (good, fair and poor) by awarding stars in each domain following the guidelines of the NOS. A “good” quality score required 3 or 4 stars in selection, 1 or 2 stars in comparability, and 2 or 3 stars in outcomes. A “fair” quality score required 2 stars in selection, 1 or 2 stars in comparability, and 2 or 3 stars in outcomes. A “poor” quality score reflected 0 or 1 star(s) in selection, or 0 stars in comparability, or 0 or 1 star(s) in outcomes.

## 3. Results

A total of 5162 titles were identified, and after excluding duplicates, 3698 titles were screened. Of these, 339 full texts were reviewed, and 335 were excluded, leaving only four articles [15,20,21,22] relevant to our study objectives and thus finally included (Figure 1). A large proportion of the studies (83% (278/335)) were excluded for having a diverse range of study aims that differed from those of our systematic review (examples of these study aims include intervention to increase HH compliance, effect on skin damage, RCT to compare various HH products and many more). The studies were conducted between 1986 and 2015; all were conducted in the United States of America (USA) (Table 1).

The studies were clinically heterogeneous due to differences in study designs (both trials and in-vitro studies), settings (e.g., health care, household and laboratory settings), participants (nurses, households and volunteers), and diagnostic methods applied. Only two studies were considered to be of good quality defined as NOS score ≥ 7 [15,20], one was of fair quality NOS score ≥ 5 [21] and the other was of poor quality NOS score < 5 [22].

Two studies involved household participants, one study involved HCWs in a neonatal intensive care unit (NICU), and the other was an experimental study that compared the AMR outcomes in vitro, in volunteers, and in pig skin. The sample size was mentioned in three studies to be 119, 224 and 238 [15,20,21], and in the experimental study the sample size was not mentioned [22].

The two studies conducted in household settings did not demonstrate that HH products increase the frequency of resistance, the study conducted in the NICU setting showed hand HH product and skin condition may influence resistance patterns of hand flora of nurses, and the other study conducted in an in-vitro setting showed that triclosan alone and triclosan containing soaps can increase resistance against *Staphylococcus aureus*.

## 4. Discussion

This systematic review provides only inconclusive evidence; two studies showing no evidence of AMR following long term use of HH products, one showing evidence of AMR and another experimental study showing only partial evidence of AMR. The discrepancy in these results may have stemmed from heterogeneity in studies. Studies conducted in household settings showed no AMR, whereas a study in a hospital setting involving HCWs in NICU showed that HH product and skin condition may influence resistance patterns of hand organisms. This probably indicates that prolonged and persistent use of HH products, as happens in health care settings, leads to resistance. On the contrary, a systematic review that examined the efficacy of HH products containing triclosan in the emergence of AMR bacteria demonstrated that prolonged use of (>1 year) of HH products led to triclosan-adapted cross-resistance among community dwellers. Additionally, compared to plain soap, triclosan did not provide added benefit in reducing infectious disease symptoms or bacterial counts on hands [14]. Similarly, in one of our included studies, the minimum inhibitory concentrations of triclosan was reported to increase several times after exposure of *S. aureus* to triclosan [22] and another study showed the emergence of AMR following the using of chlorhexidine-containing soap [21]. Decreased susceptibility of triclosan against *Mycobacterium smegmatis*, *Escherichia coli*, *Klebsiella oxytoca*, *Aranicola proteolyticus* and *Stenotrophomonas maltophilia* in various clinical samples has been reported in some studies [23,24]. Exposure of bacteria to triclosan can trigger AMR by making its membrane impermeable to triclosan and slowing down of the biochemical processes in microbes by triclosan [25]. As a result, subsequent exposure to triclosan in hand washing products has been seen to have little or no effect on microbial growth [2]. Worryingly, due to the similar regulatory mechanisms involved, microorganisms that have developed resistance to triclosan have also been resistant to antibiotics used to treat infections [26].

HH remains a key component in the suite of preventive measures used against COVID-19, and the World Health Organization (WHO) continues to endorse HH irrespective of vaccination status [27]. AMR due to excessive use of several antimicrobial agents has become one of the major concerns worldwide. Apart from antimicrobials (antibiotics, antivirals and antiparasitic agents) use of surfactants, alcohol, and hydrogen peroxides are also known to cause resistance to microorganisms [28,29,30,31]. For instance, a surveillance study conducted in two major hospitals in Melbourne, Australia over 18 years from 1997 to 2015 showed that *Enterococcus faecium* isolates resistant to alcohol were 10-fold more common in the recent decade [30]. A rise in other drug-resistant enterococcal infections has been reported from other parts of the world and Australia, and has been attributed to excessive use of alcohol-based hand sanitisers [32]. Though these reports are confined to medical/hospital settings, the role of alcohol in causing AMR in community settings and natural environments cannot be underestimated due to the recent ubiquitous use of HH products to reduce transmission of SARS-CoV-2 in the COVID-19 pandemic, which has been already highlighted in another review article [33].

This systematic review assesses for the first time the effect of HH on AMR. This remains a highly under-researched area. Even though the findings of this review are inconclusive, it is important to note that at least one study involving HCWs shows that AMR following HH is a possibility but perhaps not so in household settings. This review has a number of limitations. Despite extensive searching, only four studies were identified to meet the inclusion criteria. A further updated scoping search of MEDLINE in October 2021 revealed no more additional studies. The updated search however revealed two related studies that showed: (1) AMR bacteria still remain susceptible to hydro-alcoholic products [34], (2) a hand washing sink can also be colonised with an AMR bacterium [35]. The quality of the included studies in this review varied with only two being of good quality. Further, the duration of use of HH products was not precisely stated across the studies. Another important limitation is that there has been no published work on the topic of this review in the last 6 years. The latest of the four included manuscripts was published in 2015; this indicates a serious gap in research in this field. Another possible reason there has been no further research on this is the US Food and Drug Administration’s ruling in 2019 against the use of antibacterial agents including triclosan in consumer HH products. Another limitation is the use of fairly rigid inclusion criteria that resulted in only limited literature to be included.

## 5. Conclusions

This systematic review of four heterogeneous studies shows that prolonged use of HH products may cause AMR in health care settings, but there is no conclusive evidence that AMR occurs in household settings. The certainty of the evidence is moderate. More studies are required to understand AMR following prolonged use of HH products, and the current COVID-19 pandemic provides a natural experiment to explore the phenomenon.

## Figures and Tables

**Table 1:** Summary of the included studies.

Authors, Year [Ref]	Country	Study Year	Age	Gender	Study Type	Setting	Participants	Sample Size	Key Finding	NOS Score
Aiello et al., 2004 [20]	USA	2003	NR	NR	Double-blinded randomised intervention trial	Household	Household, the primary caregiver	224 (half received antibacterial products)	No statistically significant association between triclosan MICs and susceptibility to antibiotic was found. There was an increasing trend in the association of ORs for all species, compared at baseline (OR 0.65, CI_95%_ 0.33–1.27) versus at the end of the year (OR 1.08, CI_95%_ 0.62–1.97) and for GNB alone at baseline (OR 0.66, CI_95%_ 0.29–1.51) versus the end of year (OR 2.69, CI_95%_ 0.78 to 9.23) regardless of the hand-washing product used. There was a significantly higher proportion of *Acinetobacter lwoffii* for which the triclosan MICs were greater than the median at the end of the year compared to the baseline (*p* > 0.001). There were significantly higher proportions of *Klebsiella pneumoniae* and *S. aureus* for which the triclosan MICs were greater than the median at baseline compared to the end of the year (*p* ≥ 0.013 and *p* ≥ 0.001, respectively). There were no significant differences when we compared the proportion of triclosan MIC values greater than the median at baseline versus that at the end of the year for any of the other organisms (all *p* ≥ 0.05)	*** *** *
Aiello et al., 2005 [15]	USA	One full year, before 2005	NR	NR	Double-masked randomised home intervention trial	Household	Household primary caregiver	238	Antibacterial products did not lead to a significant increase in AMR after one year (OR 1.33, CI_95%_ 0.74–2.41)	*** *** **
Cook et al., 2007 [21]	USA	March 2001–January 2003	Averageage 41.1 years	116 female, 3 male	Sub-study of a larger cross-over clinical trial	Hospital	NICU staff nurses	119	When antiseptic soap was used, there was a significant increase in *Staphylococcus epidermidis* isolates resistant to oxacillin (RR 1.92; CI_95%_ 1.08–3.43) and gentamicin (RR, 1.50; CI_95%_ 1.00–2.27) and a 7.22 times increased risk of rifampicin resistance among *Staphylococcus warneri* isolates (CI_95%_ 2.97–17.56)	*** **
Geraldo et al., 2008 [22]	USA	Not mentioned	NR	NR	In-vitro, a volunteer method and in-vivo method	Laboratory setting	Effectiveness of soaps evaluated using an in-vitro tube dilution method, a volunteer method, and 2 pig skin methods. No specifics about volunteers have been provided	NR	The MIC and minimum bactericidal concentrations of triclosan alone and triclosan-containing soaps against *S. aureus* increased 8- to 62.5-fold after passage (20 times), whereas those of TPB and FPB (both alone and in soap) were unchanged to 62.5-fold after passage (20 times)	**

CI = Confidence Interval; FPB = Functional Pork Broth; GNB = Gram Negative Bacteria; MIC = Minimum Inhibitory Concentrations, NOS = Newcastle-Ottawa Scale; NR = Not Recorded; OR = Odds Ratio; RR = Relative Risk; TPB = Tryptose Phosphate Broth; USA = United States of America. The asterisks in the last column (NOS Score) represents number of points in NOS Scale each paper received so (**) means 2 points, (*** **) means 5 points and (*** *** *) means 7 points.

## Data Availability

Not applicable.
